# Aminoglycoside Resistance in ICUs: Are We Running out of Drugs, for Bad Bugs

**Published:** 2011

**Authors:** Mojtaba Mojtahedzadeh, Laleh Mahmoudi

Many classes of antibacterial drugs are currently available for physicians to use, however the plan of antibiotic development has slowed during the last decade.

The World Health Organization (WHO) has identified antimicrobial resistance as one of the 3 most important problems for human health. Antimicrobial resistance is a growing problem worldwide, especially in hospitals, where resistant organisms are often first detected in ICU (Intensive Care Unit). It is demonstrated that resistant bacteria are more isolated from ICUs compared with other hospital wards and outpatient clinics, and ICU stay may be an independent risk factor for acquiring multidrug-resistant strains infection. Moreover, it must be considered that ICUs patients are often colonized with multidrug- resistant strains, which often spread to other wards.

Resistance to the current antibiotics is a serious problem in the whole world including our country, Iran. New antibiotics that have been discovered and introduced into clinical practice in the last few years are mostly active against Gram-positive organisms, forcing us to consider aminoglycosides and polymixins.

As Infections caused by gram-negative bacteria are at the top causes of morbidity and mortality in critically ill patients; the knowledge of these organisms antibiotic resistance is crucial for ICU practitioners.

Recently, increased resistance has been observed against third-generation cephalosporins for Gram-negative bacilli, but aminoglycosides are a class of antibiotics that their use has been associated with less increase in microbial resistance over the years when compared with *β *lactam antibiotics. 

The aminoglycoside antibiotics have been used for treating gram-negative bacillary infections in critically ill patients in the 1940s and resistance to these agents was initially described in the 1960s. 

Nine aminoglycosides (gentamicin, tobramycin, amikacin, streptomycin, neomycin, kanamycin, paromomycin, netilmicin, and spectinomycin) are approved by the Food and Drug Administration (FDA) for clinical use in the United States. Of them, gentamicin, tobramycin, amikacin, and netilmicin are the main aminoglycosides still in use. 

The incidence of antibiotics resistance in hospital-acquired infections varies among bacterial species, clinical settings, and even countries, and may be related to local epidemic spread of a few colonies.

In some reports approximately 75% of Klebsiella pneumoniae, 87% of Enterobacter spp, 55% of Pseudomonas aeruginosa, and 75% of methicillin-resistant *Staphylococcus aureus *(MRSA) strains were drug-resistant to at least three different classes.

Although, in Gerding et al study, resistance to aminoglycosides were at the lowest level in 10 years, and only Pseudomonas aeroginosa strains exibit resistance to gentamicin. Although, in some centers, amikacin has been effective against organisms resistant to gentamicin and tobramycin.

Aminoglycoside resistance to Gram-negative bacteria has different pattern among countries. For example amikacin resistance is increasingly progressive in Turkey. In southern europe aminoglycoside resistance is higher than in central europe and northern europe. Unfortunatelly, in one study performed in Iran, the rate of aminoglycoside resistance was found to be high.

One specific organism regard to aminoglycoside resistance is Entrococci which is intrinsically resistance to low to moderate plasma level of aminoglycosides. 

There is no single solution to fight the spread of antimicrobial resistant pathogens, but multiple interventions have demonstrated potential benefit.

The corn stone of all strategies for prevention of antimicrobial resistance spread is the knowledge of physicians and other healthcare providers of the importance of the problem. 

One strategy for reduction of drug resistance is to allow “difficult-to-treat” pathogens be exposed to bactericidal drug concentrations via dose optimization.

In the case of aminoglycosides, The best parameter that determines the *in-vivo *exposure of the pathogen to serum aminoglycoside concentrations is the ratio between the peak and the minimum inhibitory concentration (MIC) (peak/MIC) of the causative gram- negative pathogen.

When the peak is 8 to 10 times greater than the MIC, the best antibiotic response is achieved. Taccone *et al*. showed that because of increased volume of distribution (Vd) in patients with severe sepsis and septic shock, an initial dose of ≥ 25 mg/kg TBW of amikacin is needed to reach therapeutic peak concentrations. 

Besides, patients generally undergo various empiric antimicrobial regimens that therefore prone them to develop antimicrobial resistance because of poorly controlled antibiotic prescription and lack of a well-defined antimicrobial treatment protocol in pre-ICU as well as ICU settings in our country. 

Since 2001, different societies have tried to draw attention to the significant lack of new antibiotics for Gram-negative pathogens. In fact, in 2004 the infectious disease society of America issued their report, Bad Bugs, No Drugs.

Unfortunately, drugs in late stage development, as well as the recently approved Doripenem, offer little advantage over already existing carbapenems for treating infections due to extended expectrum beta-lactamase producing bacteria. Thus, tigecycline and the polymixins including colistin, have been used with variable success rates .

The incidence of infections due to multiple drug resistance (MDR) acinetobacter spp. Continues to increase in our ICUs, and others with almost no agent currently under research or development pipeline.

Thus, according to CDC campaign preventive approaches includes: preventing health care-associated infections, optimizing the specific diagnosis and treatment of infections, optimizing use of antimicrobial agents including improving the choice, dose, and duration of therapy, and preventing cross-transmission of resistant pathogens.

Besides, antimicrobial susceptibility/resistance should be determined and a special antimicrobial treatment protocol should be planned based on the results for each ICU. The best resistance control requires a team wok between clinical microbiology, an infection prevention program, clinical pharmacists and clinicians.

*Mojtaba Mojtahedzadeh* *is currently working as a Professor of* *Clinical Pharmacy* *at the* *Department of Clinical Pharmacy, Faculty of Pharmacy, Tehran University of Medical Sciences, Tehran, Iran* *, and* *Department of Critical Care Medicine, Sina Hospital, School of Medicine, Tehran University of Medical Sciences, Tehran, Iran.* *He coud be reached at the following e-mail address :* *Mojtahed@sina.tums.ac.ir*

*Laleh Mahmoudi* *is currently working as a residency student of Clinical Pharmacy at the* *Department of Clinical Pharmacy ,Faculty of Pharmacy, Tehran University of Medical Sciences, Tehran, Iran* *. She coud be reached at the following e-mail ddress:L_Mahmoudi@razi.tums.ac.ir*

